# Quantifying genetic variability and genotype ranking for food quality attributes in white Guinea yam (*Dioscorea rotundata*) using a multiple trait selection index

**DOI:** 10.3389/fpls.2025.1541588

**Published:** 2025-09-23

**Authors:** Alice Adenike Olatunji, Andrew Saba Gana, Kehinde Dele Tolorunse, Ryo Matsumoto, Patrick Adebola, Asrat Asfaw

**Affiliations:** ^1^ International Institute of Tropical Agriculture (IITA), Ibadan, Nigeria; ^2^ Department of Crop Production, School of Agriculture and Agricultural Technology, Federal University of Technology Minna, Gidan Kwano, Minna, Nigeria

**Keywords:** log odds, food quality, Pearson correlation, genotype by environment interaction, multi-trait genotype–ideotype distance (MGIDI) index

## Abstract

**Introduction:**

Yield and food product quality are important attributes in the selection of yam (Dioscorea spp.) cultivars. Among yam-based food products, boiled yam and pounded yam are traditional dishes that hold cultural, nutritional, and sensory significance in regions where yam is a staple food.

**Materials and methods:**

This study evaluated the starch, sensory, and textural attributes of boiled and pounded yam products from 25 white Guinea yam (Dioscorea rotundata) genotypes grown across five environments. The sensory quality of both boiled and pounded yam products was assessed through sensory evaluation, while an instrument-based textural profile assay (ITPA) was conducted on pounded yam.

**Results and conclusion:**

Environmental influences associated with cropping seasons and locations varied across traits, ranging from 1.4% (adhesiveness) to 20.7% (cohesiveness). Significant positive correlations (p <0.001) were observed between the overall acceptability of pounded yam and its aroma, taste, color, and appearance. Crude starch was positively correlated (p <0.001) with instrumental gumminess, chewiness, and resilience, but negatively with sensory texture, highlighting the need for balanced starch content. Using the log odds ratio analysis method, TDr1741073 was identified as a superior genotype. Similarly, the multi-trait genotype–ideotype distance index (MGIDI) highlighted it as outstanding in both attributes, making it a strong candidate for use as a parent in breeding programs.

## Introduction

Yam (Dioscorea spp.) is a preferred staple food and cash crop in the humid and sub-humid tropics of Africa, where over 98% of the world’s yam production—equivalent to approximately 88 million tons—is cultivated (FAOSTAT, 2022). The genus comprises approximately 600 species, with *D. alata*, *D. cayenensis*, and *D. rotundata* being the most widely cultivated (Darkwa et al., 2019). Yams are rich in carbohydrates, proteins, minerals, and vitamins, serving as both a vital food source and an income generator for millions of people (Darkwa et al., 2019). Its cultivation is primarily subsistence-based and heavily dependent on inputs from the natural environment.

In Nigeria, particularly at institutions such as the International Institute of Tropical Agriculture (IITA), yam breeding primarily focuses on developing advanced yam varieties that meet the preferences of end-users, encompassing both farmers and consumers. This focus includes key traits such as consistent tuber quality, high tuber yield, low tuber flesh oxidation or browning, increased dry matter content, and resistance to yam mosaic virus and yam anthracnose disease (Darkwa et al., 2020; Agre et al., 2021; Asfaw et al., 2023). Farmers prioritize traits such as high germination rates, disease resistance, large tuber size, early maturity, and good pounding qualities in yam varieties (Kalu et al., 2023).

In the past 20 years, Nigeria has seen the release of several improved yam varieties (Africayam, 2023), primarily aimed at enhancing productivity and resistance to disease. Despite these advances, the full potential of these varieties has not been fully realized, primarily due to limited awareness of their food quality and consumer preferences—critical attributes that directly impacts consumer acceptance and marketability. The quality of raw yam tubers is important for the acceptability of yam food products by consumers, processors, and farmers. Consumers of pounded yam typically assess the product’s palatability based on hand feel and color before even considering its taste or flavor. Any defect in these attributes can negatively affect product acceptability by consumers (Otegbayo et al., 2023). In addition, the limited adoption of standardized phenotyping tools for sensory attributes poses further challenges (Amah et al., 2020).

According to Kohyama (2020), sensory quality encompasses a group of physical characteristics derived from how food is perceived by the sense organs. Recent efforts have aimed to characterize various yam landraces in Nigeria, primarily through farmers' perceptions of yam quality. This approach provides fundamental insights into yam food quality and its suitability for various culinary applications (Otegbayo et al., 2010). Notably, textural quality emerged as the primary food quality attribute considered by consumers of pounded yam Otegbayo et al. (2017). Texture-based assays have emerged as important tools applicable to a wide array of food products, aiding in research and product development. Texture, in the context of pounded yam, encompasses a multitude of sensory properties valued by consumers, including stretchability, smoothness, adhesiveness (stickiness), and cohesiveness (moldability) (Egesi et al., 2003; Otegbayo et al., 2017).

A better understanding of target environments is vital for a yam breeding efforts aimed at developing and identifying improved genotypes with superior productivity, tuber quality, and utilization potential (Alabi et al., 2019; Emmanuel et al., 2022). Hyman et al. (2013) and Emmanuel et al. (2022) emphasized that target environments—which consist of various farms and seasons—are often highly variable and can lead to variable phenotypic expressions of plants within a crop under cultivation. This connection between growth environment and plant phenotypic expression—commonly referred to as genotype-by-environment interaction (GEI) (Ceccarelli and Grando, 2007)—often influences the nature, magnitude, and predictability of selection in breeding program. Although GEI presents a major challenge to breeding program efficiency, it should not be disregarded; rather, it can be leveraged as an opportunity (Alabi et al., 2019; Emmanuel et al., 2022). Environmental profiling enables a strategic approach to selecting experimental sites, significantly enhancing the ability to predict the performance of yam breeding trials. In essence, by gaining a deeper understanding of the interaction between genotypes and their surrounding environments, breeders can optimize breeding efforts, ultimately leading to the development of superior yam varieties that meet the demands of producers, consumers, and other users across the value chain.

Furthermore, when devising breeding strategies, it is essential to understand the heritable variations and genetic correlations among economically significant traits, along with the expected outcomes within the breeding population (Asfaw et al., 2021). The substantial genetic variability observed in the quantitative traits of white Guinea yam underscores the potential for genetic improvement through well-structured breeding programs (Agre et al., 2023; Asfaw et al., 2021; Norman et al., 2021). Understanding this genetic diversity and the breeding potential of different genotypes is pivotal in selecting the most suitable parent stock. Thus, the objective of this study is to assess the quality attributes of selected yam genotypes for pounded and boiled yam food products using sensory and instrument-based textural methods. The study also aimed to quantify the contribution of genetic effects and genotype-by-environment interaction (GEI) patterns to the expression of quality attributes.

## Materials and methods

### Plant materials and trial establishment

Twenty-five genotypes from the International Institute of Tropical Agriculture (IITA) yam breeding program were used in this study. Details of the genotypes and testing locations are presented in **Supplementary Tables 1, 2**. The experiments were conducted at Ikenne, Abuja, and Ibadan, Nigeria, at the following coordinates: Ikenne (l6.85°N, latitude 3.69°E), Abuja (9.13°N, 7. 23°E), and Ibadan (7. 26°N, 3.54°E). The genotypes were planted using a 5 × 5 alpha lattice design, with three plants per plot at a spacing of 1 m × 1 m, and the experiment was replicated twice during the 2022 and 2023 planting seasons.

### Tuber sampling and sample preparation

The yam tubers were harvested eight months after planting and a single representative tuber per plot from the five environments (a combination of three locations and two seasons) was used for sample preparation. Each sampled tuber was divided longitudinally from the proximal to the distal end. The tuber was peeled, washed, and towel-dried. From the middle section, 100 g was used for crude starch extraction, while the remaining portions were diced into 1 m × 1 m cubes to ensure proper representation of the entire tuber for sensory and instrument-based assays. A 500 g portion of each yam sample was weighed for quality assessment, focusing on sensory attributes of both pounded and boiled yam genotypes. Additionally, an instrumental assay was conducted exclusively for the pounded yam product. Cooking and pounding were performed using the QASA machine (QYP-6000, capacity 3.6 L capacity, Qasa, Cheerfengly Industrial Co., Ltd., Taipei City, Taiwan). A total volume of 380 ml of water was measured using a measuring cup and poured into each machine pounder. The cooking time was set to 10 min for each sample, after which 80 g was collected, placed in a ziplock bag, and stored in a warmer to retain temperature. The remaining cooked yam were then pounded for 3 min in the machine. Each sample was wrapped in cling film and placed in a ziplock bag. All samples were properly labeled with a marker to avoid mix-ups and to facilitate easy identification.

### Sensory evaluation

Ten panelists, all experienced yam eaters, participated in the assessment of the boiled and pounded yam samples. Each panelist was given 5 g of coded pounded yam and boiled yam samples, one at a time, for assessment. Descriptors, as indicated in **Table 1**, were used for the evaluation.

**Table 1:** Boiled and pounded yam rating traits.

Boiled yam characteristics	Pounded yam characteristics	Rating scale
Appearance	Appearance	1 = Dislike extremely; 2 = Dislike; 3 = Neither like nor dislike; 4 = Like; 5 = Like extremely
Color	Color	1 = Dislike extremely; 2 = Dislike; 3 = Neither like nor dislike; 4 = Like; 5 = Like extremely
Aroma	Aroma	1 = Dislike extremely; 2 = Dislike; 3 = Neither like nor dislike; 4 = Like; 5 = Like extremely
Taste	Taste	1 = Dislike extremely; 2 = Dislike; 3 = Neither like nor dislike; 4 = Like; 5 = Like extremely
Texture	Texture	1 = strong; 2 = intermediate; 3 = soft
Mealiness	Mealiness	1 = soggy; 2 = slightly mealy; 3 = mealy
	Moldability	1 = not mold well/sticky at hand; 2 = intermediate; 3 = easy to mold
	Stretchability	1 = not elastic/stretch at all; 2 = intermediate; 3 = Stretch very well or very elastic

### Instrument-based texture profile analysis on pounded-yam

Textural profile analysis was conducted using the TA.XT Plus Stable Microsystems Texture Analyzer (Serial No: 2-P6-Z10447-01-V0038D577; Stable Microsystems, Godalming, UK), coupled with a compression TA-40 platen cylindrical probe. The pounded yam samples from each genotype were molded for uniformity using an open-ended, custom-made cutter plastic with dimensions of 3.6 cm × 2.2 cm. The molding process was replicated three times to ensure accuracy and reliability. Pre-test speed, test speed, and post-test speed were set at 1 mm s^−1^, 1.75 mm s^−1^, and 5 mm s^−1^, respectively. A trigger force of 10 g and a strain of 40% were used for the double compression test. The temperature of each cut sample was measured using an infrared thermometer (AD-5612A), ranging from 40°C to 55°C before placement on the TPA machine. The weight of the samples ranged approximately 25 g to 30 g. This machine simulates human chewing behavior.

### Starch extraction

A whole yam tuber was sampled and peeled, and 100 g from the middle portion was weighed and grated using a manual kitchen shredder. After shredding, the samples were transferred to a blender (IFM-C20G crush miller; Iwatani, Osaka, Japan) and fine-milled. Two hundred milliliters of water were added to facilitate blending for 2 min per sample. The samples were then poured into a 180 μm by 200 mm sieve (model no. 019-214 775-01; TokyoScreen Co. Limited, Tokyo, Japan), which was placed on a mesh for stability with a larger bowl underneath. Three liters of water was added to facilitate the washing of crude starch through the sieve, and the mixture was left for 1 h to complete the process, leaving the residue behind. The supernatant was removed, and the crude starch was air-dried and weighed after reaching a constant weight was achieved. The result was expressed as a percentage using the following formula:


$$\begin{array}{c} \text{crude starch}\left(\%\right)=(\text{weight of extracted starch}\\ ÷\text{weight offresh tubers})\;*\;100 \end{array}$$

### Statistical analysis

Data analyses were performed using various packages in the ‘R’ environment for statistical computing (R Core Team, 2022, version 4.2.2). The sensory data for boiled and pounded yam—including variables such as texture, mealiness, appearance, color, aroma, taste, stretchability, moldability, and overall acceptability—were analyzed using multinomial and ordinal logistic regression models to evaluate the effects of genotype (Geno), environment (Env), and their interactions, using the “nnet” and “ordinal” packages in R.

Multinomial logistic regression models were fitted to assess the relationship between sensory attributes and explanatory variables.


$$\text{Log}=\left(\frac{\text{p}\left(\text{yi}=1\right)}{1-\text{p}\left(\text{yi}=1\right)}\right)=\text{β}0$$

$$\text{ Log}=\left(\frac{\text{p}\left(\text{yi}=1\right)}{1-\text{p}\left(\text{yi}=1\right)}\right)=\text{β}0+\text{ β}1.\text{Envi}+\text{β}2.\text{Genoi}$$

Where:

p(yi = 1) is the probability that the response variable yi equals 1

1 − p(yi = 1) is the probability that the response variable yi equals 0

β0 is the intercept term, which represents the log-odds of the outcome yi = 1 when there are no predictors in the model.

β1 is the coefficient for the Environment (Env) predictor, which quantifies the change in the log-odds of the outcome associated with a one-unit change in the environment variable.

β2 is the coefficient for the Genotype (Geno) predictor, which quantifies the change in the log-odds of the outcome associated with a one-unit change in the genotype variable.

Envi represents the value of the environment predictor for observation.

Genoi represents the value of the genotype predictor for observation i.

For variables such as appearance, color, aroma, taste, overall acceptability, stretchability, and moldability, each coefficient (log odds) indicates the likelihood of being at or below the reference category (Meccakusa) and above it, relative to a baseline category. A positive coefficient indicates that the genotype is associated with higher log odds of receiving a lower variable rating, suggesting the genotype is more likely to be rated in the lower categories. A negative coefficient indicates that the genotype is associated with lower log odds of receiving a lower variable rating, suggesting the genotype is more likely to be rated in the higher categories. However, for texture and mealiness, the values represent regression coefficient (log odds) for each genotype relative to the reference category (Meccakusa). A positive coefficient indicates that the genotype is more likely to fall into the outcome category compared to the reference category (Meccakusa). A negative coefficient suggests the opposite.

Pearson correlation analysis was performed using the ‘metan’ package in R software version 4.1.1 (R Core Team, 2022) ‘corr_coef’ function from the package was used to plot the graph.

The multi-trait genotype ideotype distance index (MGIDI) was used to rank the genotypes based on multiple trait values, as suggested by Olivoto et al. (2019). A fixed effect model, Patterson and Williams (1976) was carried out on sensory data generated using the model:


$${\text{y}}_{\text{ijk}}=\text{m}+{\text{g}}_{\text{i}}+{\text{r}}_{\text{j}}+\text{bjk}+\text{eijk}$$

For the first stage of the MGIDI, each trait was rescaled using this model


$${\text{rX}}_{\text{ij}}=\frac{{\text{η}}_{\text{nj}}-\text{φnj}}{{\text{η}}_{0}{\text{j}}^{-\text{φ}}{\text{o}}_{\text{j}}}\text{x}\left({\text{θ}}_{\text{ij}}-{\text{η}}_{0\text{j}}\right)+{\text{η}}_{\text{nj}}$$

where the symbols indicate the following for trait j and genotype i: rXij is the rescaled two-way table; η_nj_ is the new maximum value after rescaling; φnj is the new minimum value after rescaling; η_0_j is the original maximum value; φoj is the original maximum value; and θ_ij_ is the original value for the ith genotype. Each column ranged from 0 to 100, considered the desired sense of selection (increase or decrease), and sustained the correlation structure of the original set of variables. The values obtained after rescaling for ηnj and ϕnj in a state of positive gains (ϕnj = 0 and ηnj = 100) and in a state of positive gains (φnj = 100 and η_nj_= 0) were used Olivoto et al. (2019). The second stage was to compute an exploratory factor analysis (FA) through rXij to group correlated traits into factors and then estimate the factorial scores for each row/genotype/treatment. The scores were then obtained from the data collected for the dimensionality reduction of traits and relationship structure using the following model:


$$\text{F}=\text{z}{\left({\text{A}}^{\text{T}}{\text{R}}^{-1}\right)}^{\text{T}}$$

where the letters indicate the following: F is the g × f matrix with the factorial score, Z is the g × p matrix with the rescaled means, A is the p × f matrix of canonical loading, R is the p × p correlation matrix between the traits, g is the number of genotypes, f is the factors retained, and p is the measured traits. The third stage was to compute an ideal genotype. For this, a [1 × p] vector was considered to be the ideotype matrix using the Euclidean distance between the scores of the genotypes, and the ideal genotypes were determined by the MGIDI index, as shown


$$MGIDIi=\sqrt{\sum_{\text{j}=\text{i}}^{\text{t}}{\left({\text{f}}_{\text{ij}}-\text{f}j\right)}^{2}}$$

where MGIDIi is the multi-trait genotype-ideotype distance index for the *i*th genotype, Fij is the score of the *i*th genotype in the *j*th factor (*i = 1, 2, ..., g; j = 1, 2, ..., f*), being *g* and *f* the number of genotypes and factors, respectively, and Fj is the *j*th score of the ideotype. The genotype with the lowest MGIDI is then closer to the ideotype and therefore presents desired values for all the analyzed traits.

## Results

**Figure 1:**
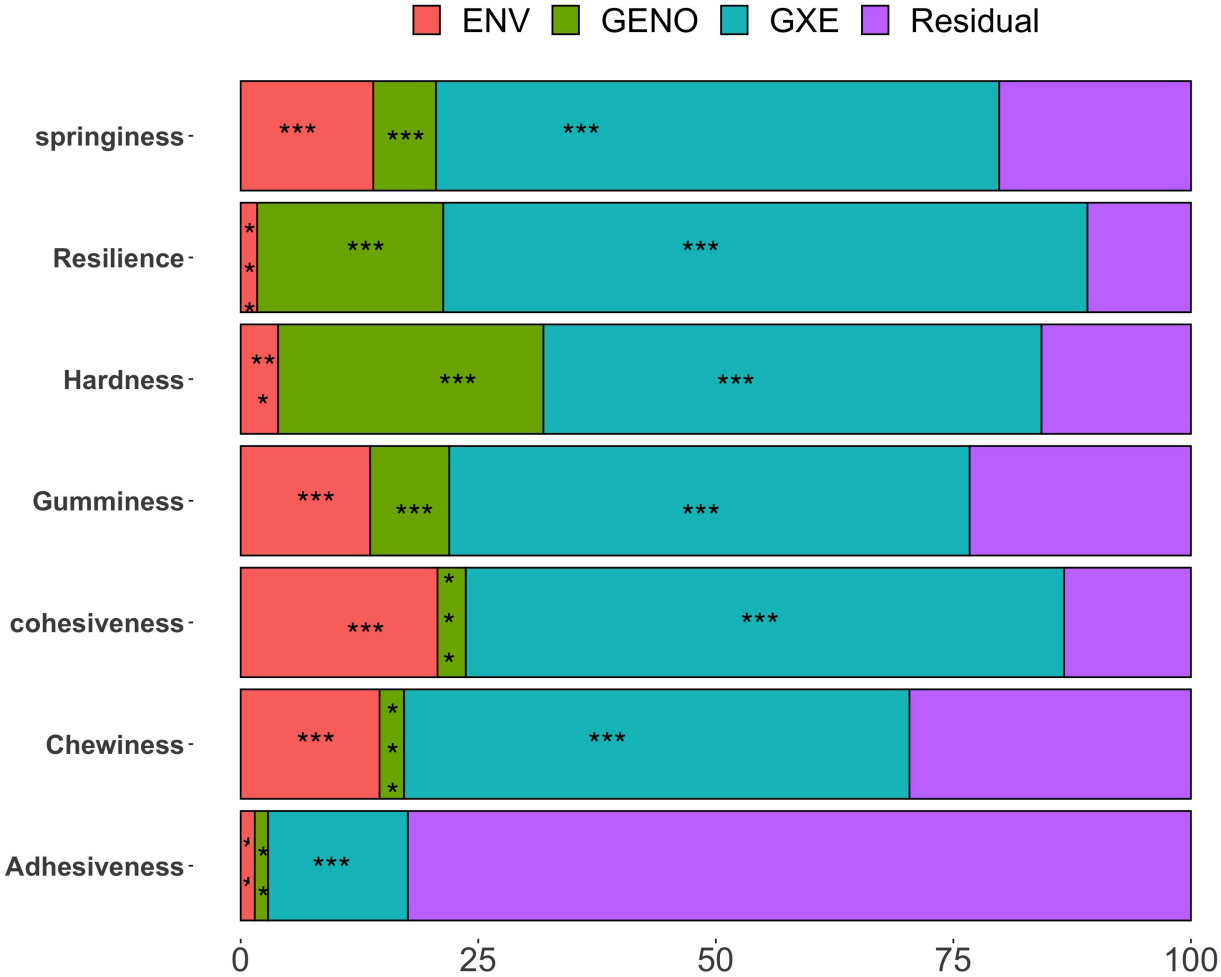
Percentage of phenotypic variance explained by each genotype, environment, genotype × environment interaction, and residual terms for ITPA quality traits on pounded-yam, sensory and crude starch traits. Significance *P ≤0.05; **P ≤0.01; ***P ≤0.001.

**Figure 1** presents the percentage of phenotypic variance explained by the genotype, environment, GEI, and residual for the quality traits assessed in the white Guinea yam genotypes, with significance levels (P values) included.

Instrument-based textural profiles showed that 1.4% (adhesiveness) to 27.9% (hardness) of the total variation was attributed to genotype. Environmental influence, associated with cropping seasons and locations, varied notably across traits, ranging from 1.4% (adhesiveness) to 20.7% (cohesiveness). A large proportion of the variation was explained by genotype × environment interaction for all traits except adhesiveness. Genotype × environment interaction ranged from 14.7% (adhesiveness) to 67.8% (resilience). The residual term, representing unexplained variation, ranged from 10.89% (resilience) to 82.4% (adhesiveness).

Geno, genotype; Env, Environment; G × E, genotype × environment; Tex_B, texture boiled yam; Mea_B, mealiness boiled yam; Appea_B, appearance boiled yam; Col_B, color boiled yam; Aroma_B, aroma boiled yam; Taste_B, taste boiled yam; Tex_P, texture pounded-yam; Mea_P, mealiness pounded-yam; Appea_P, appearance pounded-yam; Col_P, color pounded-yam; Arom_P, aroma pounded-yam; Tast_P, taste pounded-yam; Stret_P, stretchability pounded-yam; Mol_P, moldability pounded-yam; Overal_P, overall acceptability pounded-yam; Overall_B, overall acceptability boiled yam.

### Correlation among examined traits

**Figure 2:**
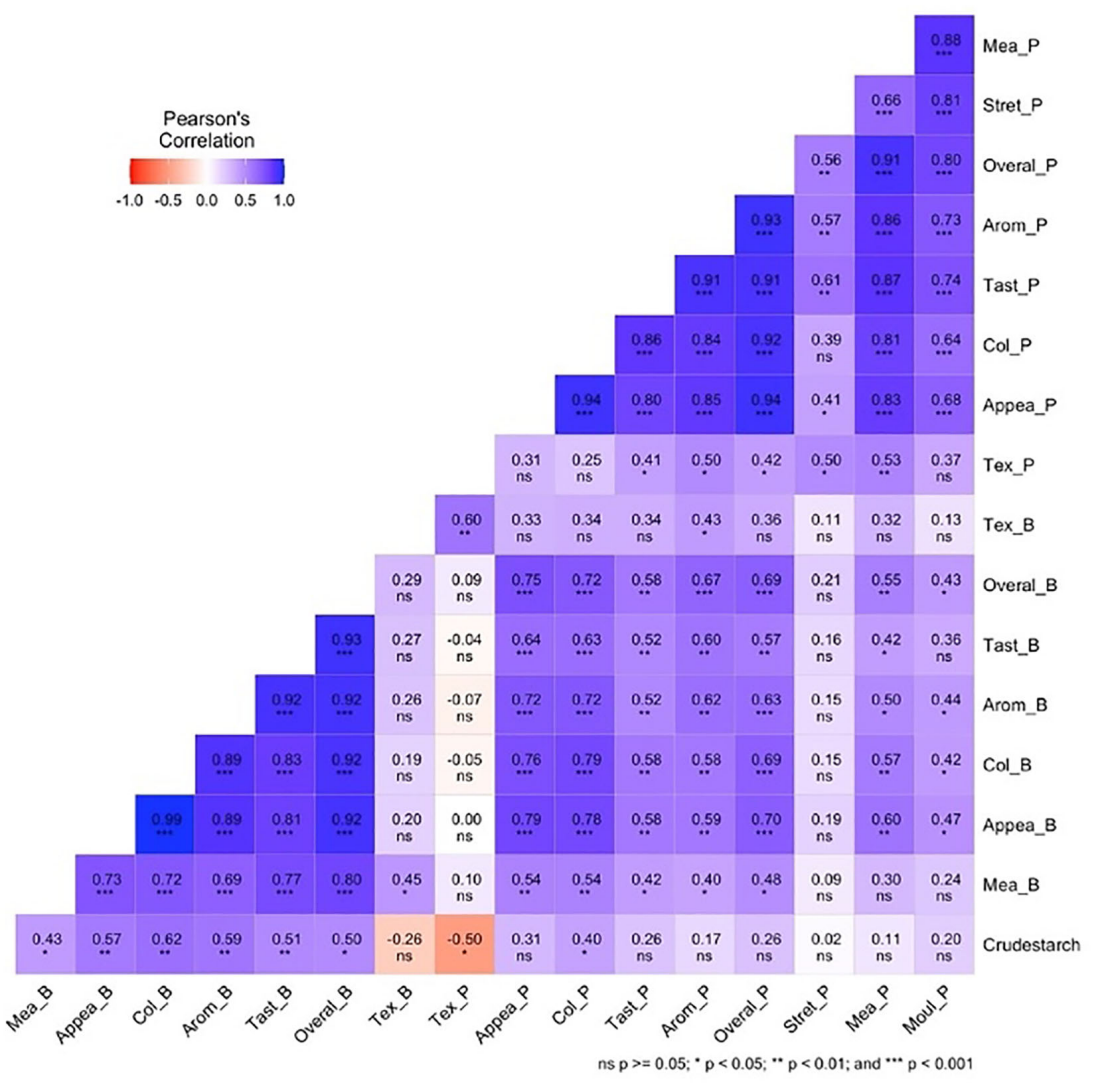
Pearson correlation plot for crude starch, pounded, and boiled traits of 25 white yam genotypes. Tex_B, texture boiled yam; Mea_B, mealiness boiled yam; Appea_B, appearance boiled yam; Col_B, color boiled yam; Aroma_B, aroma boiled yam; Taste_B, taste boiled yam, Tex_P, texture pounded-yam; Mea_P, mealiness pounded-yam; Appea_P, appearance pounded-yam; Col_P, color pounded-yam; Arom_P, aroma pounded-yam; Tast_P, taste pounded-yam; Stret_P, stretchability pounded-yam; Mol_P, moldability pounded-yam; Overal_P, overall acceptability pounded-yam; Overall_B=overall acceptability boiled yam.

The Pearson correlation plot (**Figure 2**) shows the relationships between crude starch content and various traits related to pounded and boiled yam among 25 white Guinea yam genotypes. The texture of boiled yam was positively correlated with the texture of pounded yam (r = 0.60, p <0.01). The taste of pounded yam showed a strong positive correlation with its overall acceptability (r = 0.91, P <0.001). The color of pounded yam was also strongly positively correlated with its overall acceptability (r = 0.92, p <0.001). The taste of boiled yam showed a strong positive correlation with its overall acceptability (r = 0.93, p <0.001). Crude starch was positively correlated with the appearance, color, aroma, and taste of boiled yam (r = 0.57, 0.62, 0.59, and 0.51; p <0.01) respectively. Crude starch was also positively correlated with mealiness, the overall acceptability of boiled yam, and the color of pounded yam (r = 0.43, 0.50, and 0.40; p <0.05, respectively). Crude starch was also negatively correlated with the texture of pounded yam (r = −0.50, p <0.05). Other traits were positively correlated with each other (p <0.001, p <0.05), as shown in the correlation plot, while some correlations were non-significant (**Figure 2**).

**Figure 3:**
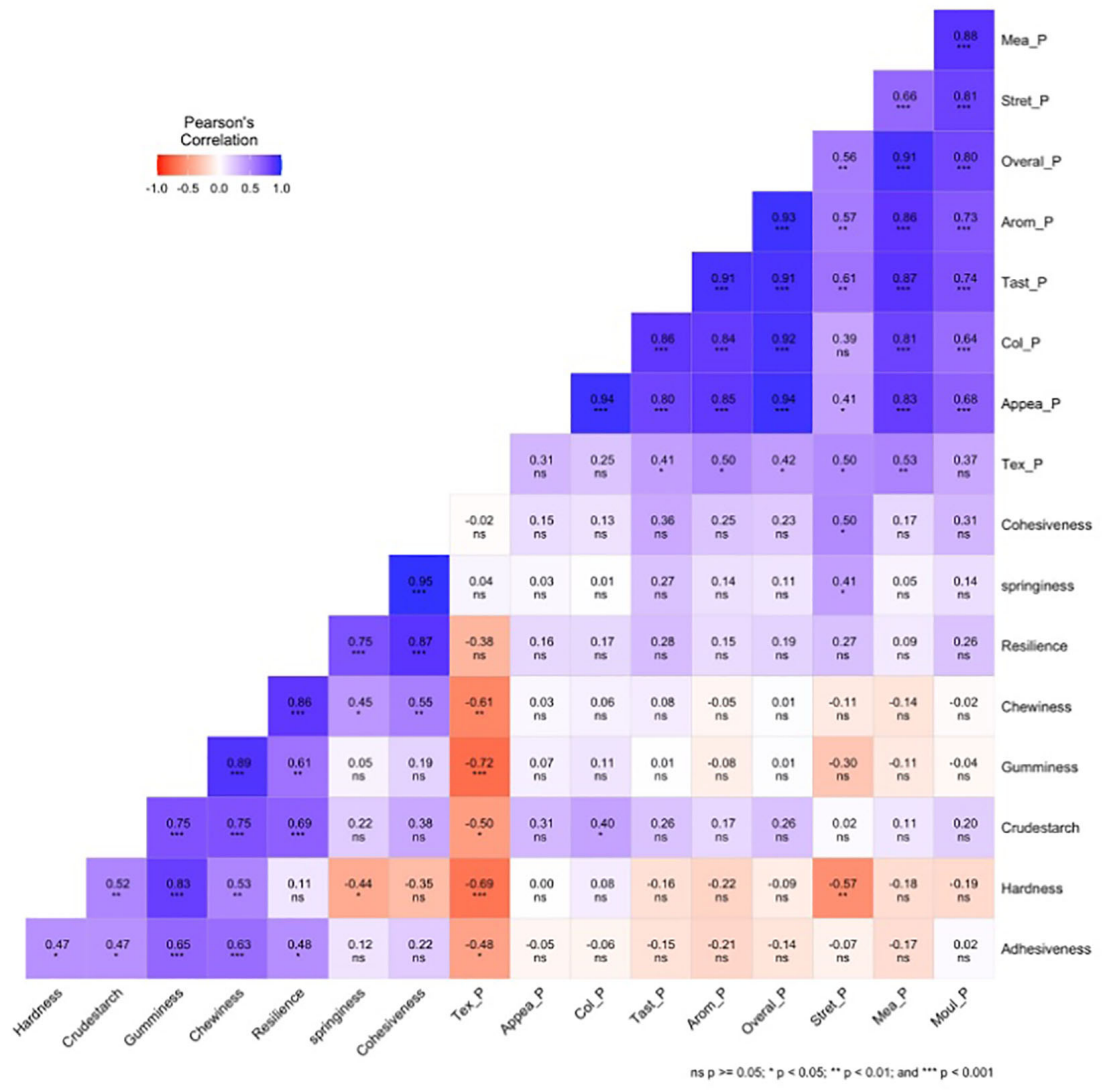
Pearson correlation coefficient among the ITPA, sensory pounded, and crude starch for 25 white yam genotypes. Tex_P, texture pounded-yam; Mea_P, mealiness pounded-yam; Appea_P, appearance pounded-yam; Col_P, color pounded-yam; Arom_P, aroma pounded-yam; Tast_P, taste pounded-yam; Stret_P, stretchability pounded-yam; Mol_P, moldability pounded-yam; Overal_P, overall acceptability pounded-yam.

The Pearson correlation coefficient between instrumental textural profile analysis, sensory attributes on pounded yam, and crude starch traits for 25 white yam genotypes across five environments (**Figure 3**) showed significance at various levels. There was a strong positive (P <0.001) correlation between the overall acceptability of pounded yam with aroma (r = 0.93), taste (r = 0.91), color (r = 0.92), and appearance (r = 0.94). There was a positive correlation between appearance and color (r = 0.94; p <0.001). Instrumental springiness was negatively correlated with hardness (p <0.05, r = −0.44) but positively correlated with chewiness (p <0.05, r = 0.45) and cohesiveness (p <0.001, r = 0.95). There was a negative correlation between stretchability and hardness (r = −0.57; p <0.01). Crude starch was positively correlated with gumminess, chewiness, and resilience (r = 0.75, 0.75, and 0.69; p <0.001, respectively). There was negative correlation between sensory texture with gumminess (r = −0.72) and hardness (r = −0.69; p <0.001). A negative correlation was observed between sensory texture and chewiness (r = −0.61; p <0.01). There was also a negative correlation between sensory texture and crude starch and adhesiveness (r = −0.50, −0.48; P <0.05, respectively). Gumminess was also positively correlated with crude starch, hardness, and adhesiveness (r = 0.75, 0.83, 0.65; (p <0.001, respectively). Some of the traits were also not significantly correlated with each other (**Figure 3**).

### Log odds ratio analysis

The Log odds ratio (LOR) results (**Table 2**) present the ordinal and multinomial logistic regression outputs for all variables assessed in the boiled yam genotype performance, with Meccakusa used as the reference genotype (LOR = 0). For the traits appearance, color, aroma, taste, and overall acceptability, each coefficient (log odds) indicates whether a genotype is at, below, or above the reference category (Meccakusa) relative to a baseline category. Both negative and positive coefficients were observed across genotypes at various significant levels, while some were not statistically significant. However, for texture and mealiness, the values represent regression coefficients (log odds) for each genotype relative to the reference category (Meccakusa). All genotypes showed positive coefficients for texture log odds, whereas for mealiness, both positive and negative log odds were observed.

**Table 2:** Log odds ratio scale for 25 boiled yam genotypes.

Boiled yam							
Genotypes	Appearance	Color	Aroma	Taste	Overall acceptability	Texture	Mealiness
TDr1716031	−0.733	−0.738	0.767	0.541	−0.037	1.825^**^	−72.943^***^
TDr1716040	−1.192***	−0.991***	−0.515	−0.195	−0.769	1.299^**^	−0.847
TDr1716069	−1.488***	−1.39***	−0.415	−0.484	−1.806***	1.098	−72.230^***^
TDr1719006	−1.928***	−1.706***	−0.723	−0.034	−1.314***	1.056^*^	−0.637
TDr1719020	−0.321	−0.107	0.291	0.463	-0.359	1.869^***^	−0.707
TDr1719029	−2.795***	−2.796***	−1.524	−1.744***	−2.446***	1.158^*^	0.479
TDr1719049	−1.072***	−1.068***	0.344	0.522	-0.754	1.627^**^	−65.725^***^
TDr1719055	−0.428	−0.334	0.956	0.446	0.058	1.846^***^	−0.472
TDr1719059	−1.361***	−1.108***	−0.822	−0.801**	−1.639***	1.122^*^	-0.921
TDr1719069	−2.378***	−2.157***	−1.468	−0.793	−2.03***	1.990^***^	−1.211
TDr1738003	0.231	0.374	0.919	0.898	0.47	1.105	−1.259
TDr1738023	−1.774***	−1.587***	−0.66	−0.956**	−1.909***	0.963	−0.105
TDr1738048	−0.136	−0.023	0.4	0.002	−0.313	1.633^**^	−0.266
TDr1738074	−0.843**	−0.73	0.588	0.446	−0.564	2.266^***^	−63.713
TDr1739023	0.038	0.16	0.589	1.086**	0.212	1.192	−1.413
TDr1739046	−0.632	−0.394	0.261	−0.121	−0.653	2.321^***^	−2.131^*^
TDr1739049	−1.031***	−0.647*	0.084	−0.198	−0.613	0.587	−2.131^*^
TDr1739058	−0.617	−0.128	0.864	0.408	−0.439	1.294^**^	−68.403^***^
TDr1739067	−1.377***	−0.929**	−0.043	−0.283	−1.21***	1.617^***^	−2.273^*^
TDr1739068	−1.772***	−1.748***	−0.23	−0.623	−1.597***	1.218^*^	−0.217
TDr1741021	−2.486***	−2.349***	−0.461	−0.111	−1.396	1.680^***^	2.094*
TDr1741042	0.182	0.187	0.471	0.524	0.056	2.106^***^	−1.15
TDr1741044	−0.575	−0.244	0.651	0.927**	−0.033	0.899	−1.925
TDr1741073	0.904	1.143***	1.442	1.417***	0.986**	1.138	−64.205^***^

Significant level, *P <0.1, **P <0.05, ***P <0.01.

For appearance, genotypes such as TDr1741073 (LOR = 0.904) and TDr1738003 (LOR = 0.231) had positive log odds, while genotypes like TDr1719029 (LOR = −2.795***) and TDr1719069 (LOR = −2.378***) showed significantly negative values.

For taste, TDr1741073 (1.417***), TDr1739023 (1.086**), and TDr1741044 (0.927**) were top performers, whereas TDr1719029 (−1.744***) and TDr1738023 (−0.956**) recorded low LORs. In terms of texture, most genotypes performed well, with TDr1739046 (2.321*), TDr1738074 (2.266***), and TDr1741042 (2.106***) exhibiting high and significant LORs.

For the pounded yam, the log odds ratio (**Table 3**) present the ordinal and multinomial logistic regression results for all the variable assessed on the pounded yam genotypes. For variables such as appearance, color, aroma, taste, moldability, stretchability, and overall acceptability, each coefficient (log odds) indicates the likelihood of being at or below the reference category (Meccakusa), or above it, relative to a baseline category. Both positive and negative coefficients were observed across the variables. However, for texture and mealiness, the values represent regression coefficients (log odds) for each genotype relative to the reference category (Meccakusa). Both positive and negative coefficients were observed at various levels of statistical significance.

**Table 3:** Log odds ratio scale for 25 pounded yam genotypes.

Pounded yam									
Genotypes	Appearance	Color	Aroma	Taste	Moldability	Stretchability	Overall acceptability	Texture	Mealiness
TDr1716031	−1.065***	−0.653	−0.406	−1.145***	−1.622***	−1.226***	−1.084***	2.066^*^	-0.309
TDr1716040	−2.135***	−1.237***	−0.947***	−0.95***	−1.083***	−0.844**	−1.647***	2.972^***^	0.399
TDr1716069	−2.501***	−1.828***	−1.859***	−1.831***	−2.241***	−1.609***	−2.783***	4.048^***^	1.238
TDr1719006	−1.568***	−1.248***	−0.385	−0.823**	−0.754	−0.09	−1.564***	2.425^**^	0.446
TDr1719020	−0.181	0.498	0.115	0.197	0.391	0.074	−0.002	0.904	-13.035^***^
TDr1719029	−2.14***	−1.849***	−1.381***	−2.05***	−1.572***	−0.48	−2.294***	2.066^*^	1.292
TDr1719049	−1.071***	−0.252	−0.466	−0.532	−0.639	0.121	−0.98***	2.069^*^	0.214
TDr1719055	−0.67	−0.076	0.255	−0.104	−0.584	0.587	−0.401	−8.865	0.009
TDr1719059	−1.307***	−0.336	−0.489	−0.304	−0.448	0.471	−0.76**	1.858	-14.684^***^
TDr1719069	−2.825***	−1.903***	−0.834**	−1.118***	−1.796***	−0.269	−2.265***	1.906	1.338
TDr1738003	−1.154***	−0.803**	−0.754**	−1.353***	−1.44***	−1.362***	−1.38***	2.348^**^	0.937
TDr1738023	−2.76***	−1.847***	−1.461***	−1.793***	−1.348***	−0.763**	−2.398***	1.782	0.224
TDr1738048	−1.474***	−1.19***	−1.156***	−1.427***	−0.576	−0.106	−1.512***	2.033^*^	0.167
TDr1738074	−1.2***	−0.375	−0.437	−0.686	−0.797	−1.133***	−1.192***	1.831	-0.211
TDr1739023	−0.836**	−0.068	−0.913**	−0.958***	−1.554***	−0.847**	−1.35***	1.301	-0.168
TDr1739046	−0.861**	−0.117	−0.448	−0.509	−0.206	0.177	−0.694**	0.709	-15.026^***^
TDr1739049	−1.03***	−0.097	−0.28	−0.648	−1.347***	−1.015***	−0.831**	0.709	-15.681^***^
TDr1739058	−1.39***	−0.59	−0.117	−0.68	−0.834	−0.363	−0.952***	3.424^***^	-15.386^***^
TDr1739067	−1.166***	−0.406	−0.731**	−1.019***	−1.511***	−1.192***	−1.107***	2.394^**^	-1.635
TDr1739068	−1.993***	−1.329***	−0.745**	−1.511***	−1.539***	−0.721	−2.329***	2.536^**^	-0.772
TDr1741021	−2.363***	−1.963***	−0.792**	−1.005***	−1.105***	−0.298	−1.759***	2.053^*^	0.266
TDr1741042	−0.385	0.367	0.046	−0.125	−0.914**	−0.781**	−0.34	1.775	-1.023
TDr1741044	−1.494***	−0.941***	−0.328	−0.817**	−0.41	0.204	−0.93***	3.195^***^	-0.165
TDr1741073	−0.019	1.172***	0.192	0.169	0.014	0.723	0.249	1.704	1.075

Significant level, *P <0.1, **P <0.05, ***P <0.01.

For appearance, genotypes such as TDr1741073 (LOR = −0.019) and TDr1719020 (−0.181) had comparatively higher (less negative) log odds, whereas TDr1738023 (−2.76*) and TDr1719069 (−2.825***) recorded significantly negative values. For color, TDr1741073 (1.172***) was the only genotype with a significant positive LOR, while most others, such as TDr1738023 and TDr1716069, had strongly negative coefficients. Texture was a strong differentiator, with top-performing genotypes including TDr1716069 (4.048***), TDr1739058 (3.424***), and TDr1741044 (3.195***), while TDr1719055 (−8.865) showed a large negative value. For mealiness, most genotypes had negative or extreme values; TDr1719020 (−13.035***), TDr1739046 (−15.026***), and TDr1739049 (−15.681***) recorded particularly poor performance. In contrast, TDr1719069 (1.338) and TDr1719029 (1.292) showed the highest positive LORs for mealiness.

Significance levels for boiled and pounded yam traits (p <0.1, p <0.05, p <0.01) are included to indicate the strength of evidence for differences in trait ratings relative to the reference genotype.

**Figure 4:**
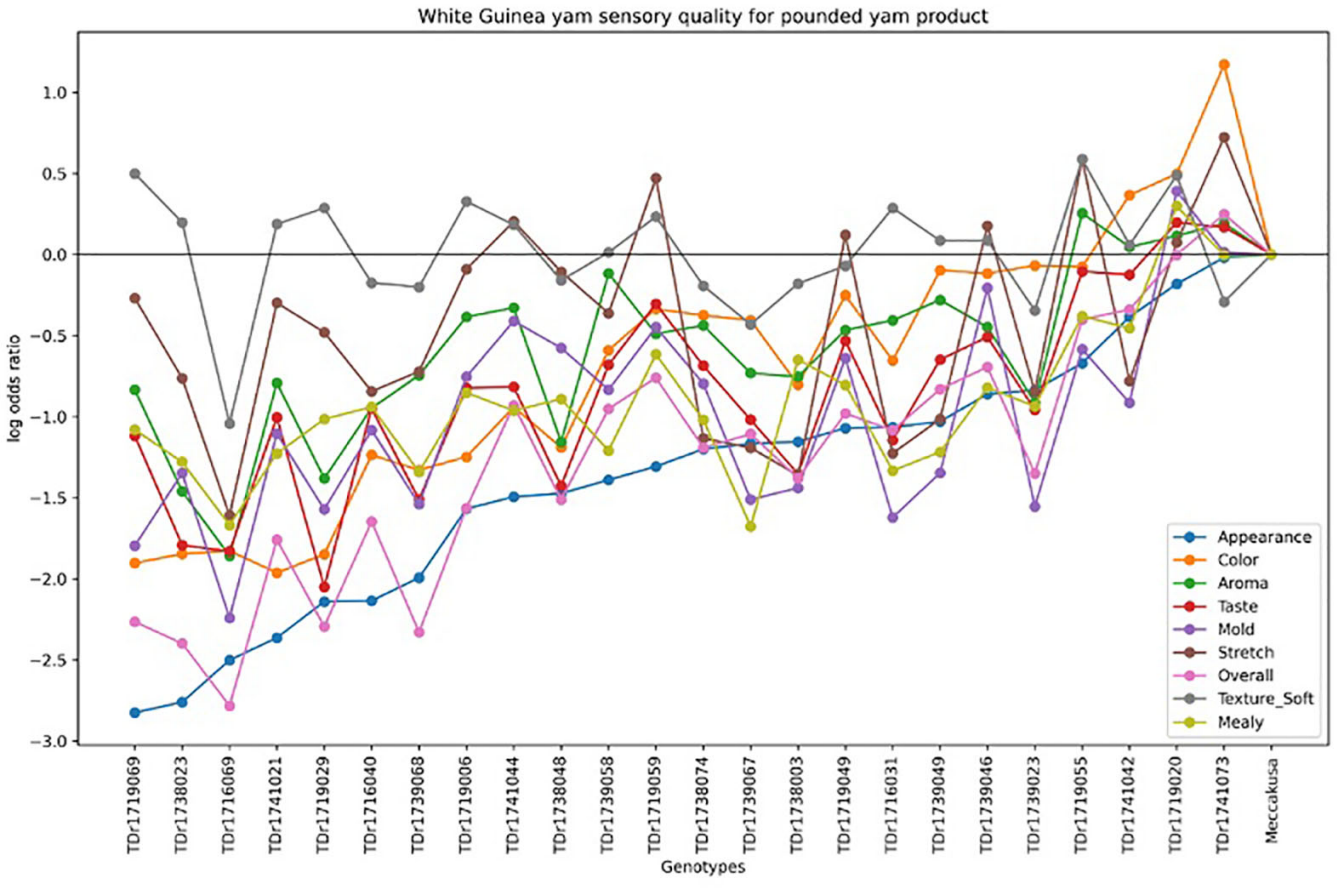
Pounded yam sensory overall rating scale pattern for 25 white yam genotypes.

**Figure 4** illustrates the variation in sensory quality among yam genotypes evaluated for pounded yam. The x-axis represents the different genotypes, with Meccakusa as the baseline reference, while the y-axis displays the log-odds ratio, reflecting the likelihood of a sensory attribute being perceived in each genotype relative to the baseline. The different colors correspond to the sensory attributes measured for pounded yam quality. The log-odds ratios varied across genotypes, highlighting differences in sensory qualities. Among the genotypes, TDr1741073 was perceived as having the best sensory quality for pounded yam compared to the baseline, Meccakusa.

**Figure 5:**
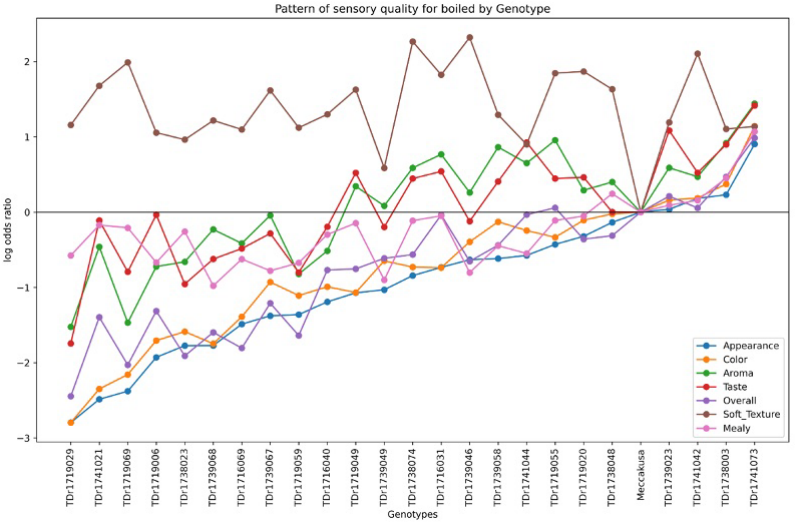
Boiled sensory overall rating scale pattern for 25 white yam genotypes.

Similarly, **Figure 5** presents the sensory quality of white Guinea yam genotypes for boiled yam. The different colors represent the sensory attributes measured for boiled yam across the genotypes. The log-odds ratios varied, with genotypes showing values above zero being preferred for sensory attributes compared to the baseline, Meccakusa. The results indicate that TDr1741073, TDr1738003, TDr1741042, and TDr1739023 exhibited superior sensory quality for boiled yam relative to other genotypes, using Meccakusa as the reference.

### Factor analysis and selection-based on multi-trait genotype–ideotype distance index

#### Instrument-based quality traits

The MGIDI index identified two factors based on the seven instrumental quality traits evaluated for pounded yam. Factor analysis (FA1) was associated with hardness, adhesiveness, gumminess, chewiness, and resilience, while factor analysis (FA2) was associated with springiness, cohesiveness chewiness, and resilience. The average communality and uniqueness accounted for 91% and 8% of the total genetic variability in the data, respectively (**Table 4**). Of the seven traits evaluated, six showed a desired selection gain using the MGIDI index, while Hardness showed an undesired selection gain. A total genetic gain of 9.49% was observed for the assessed traits where increases were desired, and 5.62% where decreases were desired (**Table 4**).

**Table 4:** Factorial loadings, communalities, uniqueness, and predicted selection gains (PSG) based on the multi-trait genotype–ideotype distance index.

Instrumental textural profile analysis traits							
Variable	FA1	FA2	Communality	Uniquenesses	Goal	PSG	Sense
Hardness	**0.87**	0.42	0.94	0.06	0	5.62	decrease
Adhesiveness	**−0.77**	0.15	0.62	0.38	100	−1.41	increase
Springiness	0.07	**0.97**	0.95	0.05	100	9.9	increase
Cohesiveness	−0.06	**0.99**	0.99	0.01	100	6.77	increase
Gumminess	**−0.97**	0.12	0.96	0.04	100	13.8	increase
Chewiness	**−0.84**	**0.51**	0.96	0.04	100	17	increase
Resilience	**−0.52**	**0.83**	0.96	0.04	100	10.9	increase
Average			0.911	0.089			

Boiled traits						
Variable	FA1	Communality	Uniquenesses	Goal	PSG	Sense
Texture	**0.76**	0.15	0.85	0	2.85	decrease
Mealiness	**−0.86**	0.72	0.28	100	2.00	increase
Appearance	1.3	0.87	0.13	100	5.45	increase
Colour	1.31	0.87	0.13	100	4.67	increase
Aroma	1.34	0.91	0.09	100	2.37	increase
Taste	1.28	0.86	0.14	100	3.32	increase
Overall acceptability	1.28	0.93	0.07	100	4.30	increase
Average		0.76	0.24			

Pounded yam traits							
Pounded yam traits	FA1	FA2	Communality	Uniquenesses	Goal	PSG	Sense
Texture	0.11	**0.76**	0.58	0.42	100	**−**1.04	decrease
Mealiness	**−0.81**	**−0.53**	0.93	0.07	100	0.904	increase
Mouldability	**−0.65**	**−**0.6	0.79	0.21	100	0.767	increase
Stretchability	**−**0.28	**−0.84**	0.79	0.21	0	**−**1.43	increase
Appearance	**−0.95**	**−**0.14	0.92	0.08	100	5.48	increase
Colour	**−0.97**	**−**0.09	0.95	0.05	100	6.14	increase
Taste	**−0.84**	**−**0.42	0.88	0.12	100	2.27	increase
Aroma	**−0.84**	**−**0.41	0.88	0.12	100	1.73	increase
Overall acceptability	**−0.92**	**−**0.36	0.97	0.03	100	5.79	increase
Average			0.85	0.15			

The bold numbers denotes higher contributing factors in each PCs as explained in the text.

**Figure 6:**
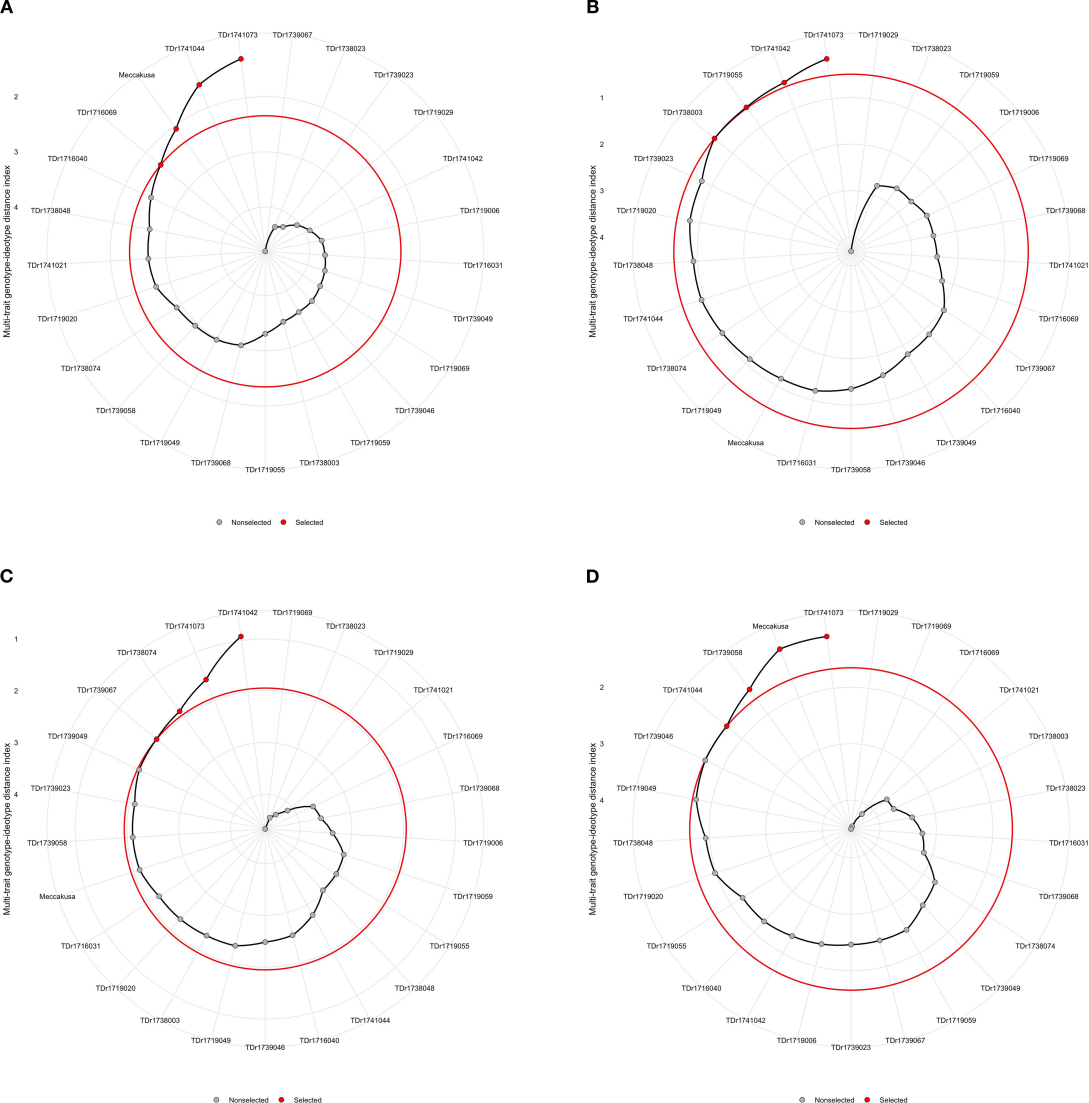
**(a–d)** Genotypes ranking and selection using the factor analysis and MGIDI index. The selected genotypes are shown in red and the unselected in black circles. The circle represents the cut-point according to the selection pressure.

Based on the MGIDI index for instrumental textural profile analysis traits, four genotypes (TDr1741073, TDr1741044, TDr1716069 and Meccakusa) were identified as high-performing genotypes for multiple traits assessed on 25 white Guinea yam genotypes (**Figure 6a**, **Supplementary Table 3**).

### Boiled yam traits

The MGIDI index identified one factor based on boiled yam traits evaluated. Factor analysis one was associated with texture and mealiness. The average communality and uniqueness accounted for 76% and 24% of the total genetic variability in the data, respectively (**Table 4**). Of the boiled traits evaluated, six showed desired selection gain using the MGIDI index. A total genetic gain of 22.11% was observed for the assessed multi-traits where increases were desired, and 2.85% for those where a decrease was desired (**Table 4**).

Based on the MGIDI index for crude starch traits, four genotypes were also identified (TDr1741073, TDr1741042, TDr1719055, and TDr1738003) as high-performing genotypes for multiple traits assessed across 25 white yam genotypes (**Figure 6b**, **Supplementary Table 4**).

### Pounded yam traits

The MGIDI index identified two factors based on the pounded yam traits evaluated. Factor Analysis one was associated with all pounded yam traits except stretchability and texture. FA2 was associated with texture and stretchability. The average communality and uniqueness accounted for 85% and 15% of the total genetic variability in the data, respectively (**Table 4**). Pounded yam traits showed desired selection gain using the MGIDI index. Increases are desired for the assessed multi-traits, with a total genetic gain of 21.65% and −1.04% for traits where a decrease was desired.

Based on the MGIDI index, four genotypes were also identified (TDr1741042, TDr1741073, TDr1738074, and TDr1739067) as high-performing genotypes for multiple traits assessed across 25 white yam genotypes (**Figure 6c**; **Supplementary Table 5**).

Combining the characteristics of sensory and instrument-based traits, the MGIDI identified four genotypes—TDr1741073, TDr1739058, Meccakusa, and TDr1739046—as best performing genotypes. Hence, the TDr1741073 genotype was the top performer across multiple traits among the 25 genotypes tested in six different environments (**Figure 6c**).

## Discussion

In this study, we assessed the genetic potential of 25 genotypes of white Guinea yam (Dioscorea rotundata) for its food product quality across five environments. Understanding the genetic variability and correlations among fresh root, color, and textural quality traits of yam and pounded yam products is a critical step toward their genetic improvement. The genotypes characterized in this study displayed large phenotypic variation for most of the evaluated traits. Some variables, such as adhesiveness and cohesiveness, showed low variability, while others, including chewiness, gumminess, and hardness, exhibited more substantial variability with positively skewed distributions. The boiled and pounded yam results demonstrated a wide range of quality attributes among the different genotypes. This substantial variation observed in both boiled yam and pounded yam underscored significant genetic variability. These results align with the findings of Asfaw et al. (2023), who also reported similar variation in food quality traits of boiled and pounded yam.

The Pearson correlation illustrates the relationship between crude starch content and various food product quality traits in white yam genotypes, providing insight into how sensory and textural attributes are impacted by tuber starch. Boiled yam texture positively correlates with pounded yam texture, indicating that genotypes with good boiled yam texture are likely to produce good pounded yam quality. Taste is crucial for consumer satisfaction, with strong correlations between taste and overall acceptability. Higher starch content enhances several sensory attributes of boiled yam but negatively impacts the texture of pounded yam. Additionally, crude starch content shows positive correlations with gumminess, chewiness, and resilience, contributing to a more robust eating experience. These findings are consistent with previous studies, such as Bakare et al. (2018), which reported significant correlations between appearance, sensory texture, and taste, emphasizing the essential role of sensory properties in determining culinary quality. A significant correlation between springiness and cohesiveness also supports the findings of Analia et al. (2011).

The log odds ratio reflected the results of ordinal and multinomial logistic regression for all variables assessed on boiled and pounded yam genotypes. For appearance, genotype TDr1716031 had a negative estimate (log odds = −0.77) suggesting lower odds of being in a lower appearance category. This indicates a tendency towards the lower category, but the likelihood of being in higher category was not statistically significant. Genotype TDr1716040, with a negative significant coefficient, showed a strong association with higher appearance ratings and reduced odds of being in a lower category, statistically significant at p <0.001. Genotypes with a positive but non-significant coefficient suggest a tendency toward lower appearance categories. This trend also applies to other variables. For texture and mealiness, genotypes with statistically positive coefficient values were more likely to be rated in higher categories compared to the reference genotype. Genotypes with positive but non-significant coefficients tended toward higher categories of the variable. This pattern was also observed for mealiness.

This can be said that the log odds ratios revealed how different genotypes compare to the reference category “Meccakusa,” in terms of sensory attributes. Significant coefficients indicate strong associations, whereas non-significant one suggests weaker or inconclusive effects. This approach aligns with the methods described by Goodman (1983) and Richard (2007), who applied log-linear models for odds in cross-classification analyses. These models offer an alternative to frequency-based methods and are also applied in participatory on-farm trial analysis. Additionally, the frequency plots show diversity in sensory qualities among the genotypes for both pounded and boiled yam. TDr1741073 was most preferred over other genotypes compared to the baseline variety, Meccakusa, which is visually represented by the horizontal reference line at a log odds ratio of zero. Genotypes consistently showing negative log odds for attributes such as texture and mealiness suggest that these attributes are less favorable in those genotypes. The observed variability can help identify which yam genotypes are preferable for specific sensory qualities, potentially guiding breeding programs to enhance desirable sensory traits—similar to findings reported by Richard (2007).

Breeders have endeavored to incorporate desirable traits into new genetic combinations to cultivate high-performing genotypes. To achieve this, various selection indices and multi-trait methods have commonly been employed to group traits and select superior genotypes (Fatoumata et al., 2024). However, pinpointing the ideal genotype remains challenging when multiple characteristics are considered. To address this complexity, the MGIDI was used to rank white Guinea yam genotypes based on extensive data from multi-trait evaluations. The MGIDI is a powerful tool for identifying genotypes with superior overall performance and desirable genetic gains, while also facilitating the assessment of strengths and weaknesses among selected genotypes (Singamsetti et al., 2023). Twelve genotypes were selected, four from ITPA traits (TDr1741073, TDr1741044, TDr1716069, and Meccakusa), four from boiled yam sensory traits (TDr1741073, TDr1741042, TDr1719055, and TDr1738003), and four from pounded yam sensory traits (TDr1741042, TDr1741073, TDr1738074, and TDr1739067) (**Figure 6**). Genotypes with lower MGIDI values are considered to exhibit better performance and stability for the traits assessed. Focusing on these selected genotypes can enhance yam improvement program by boosting both yield and quality. Notably, TDr1741073 was selected for both boiled and pounded yam traits, indicating its favorable qualities and potential use as a parent for further improvement efforts. The MGIDI model has been applied across various crops, including white Guinea yam genotypes (Norman et al., 2022), *D. praehensilis* (Adeyinka et al., 2023), wheat (Meier et al., 2021), and eggplant genotypes (Uddin et al., 2021).

## Conclusion

This study assessed 25 genotypes of white Guinea yam for food product quality across five environments, revealing significant variability across all evaluated traits. This variability underscores the potential for genetic improvement through targeted breeding programs. Importantly, genotype performance varied significantly across environments, highlighting the influence of environmental factors on trait expression. The multi-trait genotype ideotype distance index (MGIDI) emerged as a valuable tool, identifying four genotypes, each with superior performance for boiled, pounded yam, and instrumental textural profile analysis (ITPA) traits. These findings underscore the importance of multilocational testing of yam genotypes to account for environmental influences and optimize selection strategies for improved genotypes.

## Data Availability

The raw data supporting the conclusions of this article will be made available by the authors, without undue reservation.
